# Detection of Chronic Wasting Disease Prions in Raw, Processed, and Cooked Elk Meat, Texas, USA

**DOI:** 10.3201/eid3102.240906

**Published:** 2025-02

**Authors:** Rebeca Benavente, Fraser Brydon, Francisca Bravo-Risi, Paulina Soto, J. Hunter Reed, Mitch Lockwood, Glenn Telling, Marcelo A. Barria, Rodrigo Morales

**Affiliations:** The University of Texas Health Science Center at Houston, Houston, Texas, USA (R. Benavente, F. Bravo-Risi, P. Soto, R. Morales); University of Edinburgh, Scotland, UK (F. Brydon, M.A. Baria); Universidad Bernardo O’Higgins, Santiago, Chile (F. Bravo-Risi, P. Soto, R. Morales); Texas Park and Wildlife Department, Kerrville, Texas, USA (J.H. Reed, M. Lockwood); Colorado State University, Fort Collins, Colorado, USA (G. Telling)

**Keywords:** Chronic wasting disease, prions and related diseases, CWD, Creutzfeldt-Jakob disease, CJD, bovine spongiform encephalopathy, BSE, Texas, United States

## Abstract

We describe chronic wasting disease (CWD) prion detection in raw and cooked meat from a CWD-positive elk. We found limited zoonotic potential in CWD prions from those meat products. Nonetheless, risk for transmission to humans is still unclear, and monitoring of circulating and emerging CWD prion strains for zoonotic potential is warranted.

Prion diseases cause various diseases that affect several animal species, including scrapie in sheep and goats (*1*), Creutzfeldt-Jakob disease (CJD) in humans (*2*,*3*), bovine spongiform encephalopathy (BSE) in cattle (*4*), and chronic wasting disease (CWD) in cervids (*5*). In the 1990s, several atypical CJD cases occurred among persons who ingested cattle-derived products infected with BSE. Those cases later were attributed to the emergence of a new human prion strain templated by BSE prions (*6*). Subsequent studies have been conducted to investigate the zoonotic potential of other prionopathies, including CWD (*7*,*8*). Although no cases of CWD transmission to humans have been reported, the potential for human infection is still unclear because contradictory results have been reported from studies in animal models, in vitro systems, and nonhuman primates (*8*,*9*).

CWD prions have been detected in the muscle of both farmed and wild deer (*10*), and at concentrations relevant to sustain disease transmission (*11*). CWD prions have also been identified across several cervid species and in multiple tissues, including lymph nodes, spleen, tongue, intestines, adrenal gland, eyes, reproductive tissues, ears, lungs, and liver, among others (*12*–*14*). Those findings raise concerns about the safety of ingesting processed meats that contain tissues other than skeletal muscle (*15*) (Appendix). In addition, those findings highlight the need for continued vigilance and research on the transmission risks of prion diseases and for development of new preventative and detection measures to ensure the safety of the human food supply. Considering that humans consume products from ≈7,000–15,000 CWD-infected cervids each year (Appendix), the need for clarification of transmission risk for prion diseases is imperative. We investigated detection of CWD prions in seasoned and unseasoned raw and cooked meats prepared from a hunter-harvested elk. We also assessed the potential for CWD prions within meat products to template the misfolding of human cellular prion protein (PrP^C^).

## The Study

We obtained different unprocessed and processed meats from a hunter-harvested, CWD-infected elk that encoded both methionine and leucine polymorphic variations at prion protein (PrP) position 132. We used meat and meat-derived products, including filets, jerky steak cuts, hamburger meat, chili meat, sausage, ham, cutlets, and boneless steaks (Appendix Figure 1). The meat was prepared from a 5-year-old bull elk (*Cervus elaphus nelsoni*) that was positive for PrP^Sc^ (scrapie isoform of the prion protein) in the obex region of the brain; lymph node was not tested. The elk was harvested on December 10, 2020, and the sample was confirmed as CWD-positive January 8, 2021. The animal was collected in Medina County, Texas, on a private high-fenced hunting ranch. 

**Figure 1:**
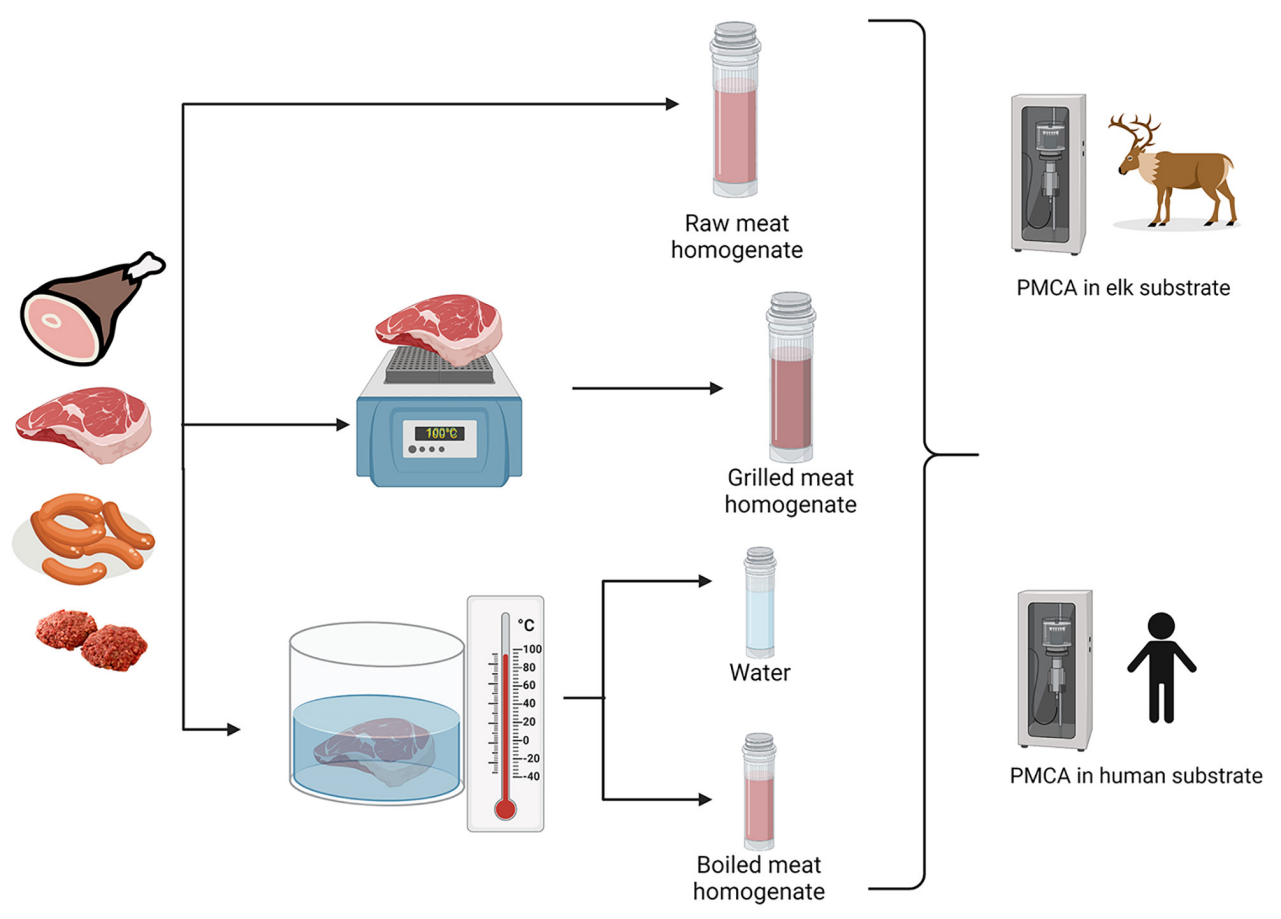
Flowchart of experimental strategy to detect chronic wasting disease prions in raw, processed, and cooked elk meat. We sampled ham, boneless steak, sausage, and hamburger meat from an elk that tested positive for chronic wasting disease. We homogenized raw meat samples and samples of meat cooking by grilling or boiling. We then tested homogenates of raw and cooked meat and the remnant water used in the boiling procedure by PMCA using elk and human substrates. PMCA, protein misfolding cyclic amplification.

**Figure 2:**
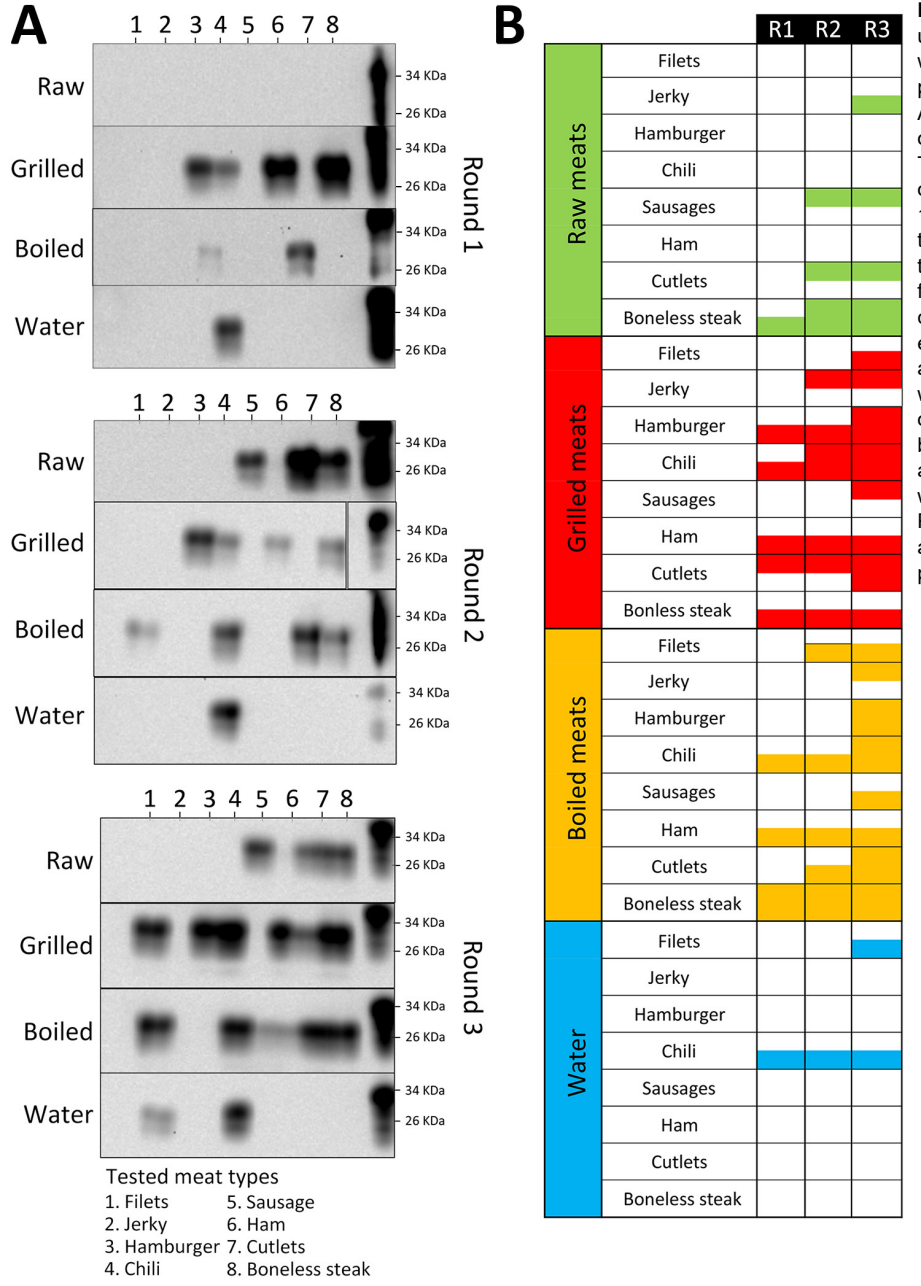
Western blot of PMCA used for detection of chronic wasting disease prions in raw, processed, and cooked elk meat. A) Representative PMCA data of the different samples tested. The panels depict the results obtained in 3 PMCA rounds of 1 of the replicates conducted in this analysis. All samples were treated with proteinase K, except for PrP^C^ that was used as a control for antibody activity and electrophoretic mobility. Numbers at the right depict molecular weight markers. B) Summary of the PMCA detection data in both replicates for raw, grilled, and boiled meats and for the water used in the boiling process. PMCA, protein misfolding cyclic amplification; PrP^C^, cellular prion protein; R, round.

We tested raw meat samples for CWD prions by using the protein misfolding cyclic amplification (PMCA) technique in an elk substrate (Figure 1). We selected the elk PMCA substrate to maintain the prion protein sequence homology between PrP^C^ and the suspected PrP^Sc^ in meats. In a first PMCA round, prion detection was negative for most of the raw meat samples, except the boneless steak, which had a positive PMCA signal in 1 of the replicates (Figure 2). To increase the sensitivity of prion detection, we performed 2 additional PMCA rounds (Appendix Figure 2). In a second PMCA round, we observed positive signals for additional specimens, including sausages and cutlets (Figure 2). The jerky sample also provided CWD prion signals in a third PMCA round (Figure 2). No other samples tested PMCA-positive in that analysis.

To test persistence of CWD prions in cooked meat products, we grilled and boiled different pieces of the meat types, mimicking a medium-well cooking status by considering the external and internal appearance and internal temperatures (Appendix Table). Of note, grilling substantially increased prion detection by PMCA, and 5 sample meat types, hamburger, chili meat, ham, cutlets, and boneless steak, were positive in a first PMCA round (Figure 2). We observed a positive PMCA signal for jerky meat in a second PMCA round and an increased number of positive replicates for the samples detected in the first round. At the third PMCA round, all grilled and boiled meats were positive for CWD prion in >1 replicate, strongly suggesting that grilling increased the availability of CWD prions for in vitro prion amplification (Figure 2). We observed similar results when we boiled different cuts of the same specimens. Of note, the water used to boil some of the meat samples was also positive by PMCA analyses (Figure 2; Appendix).

**Figure 3:**
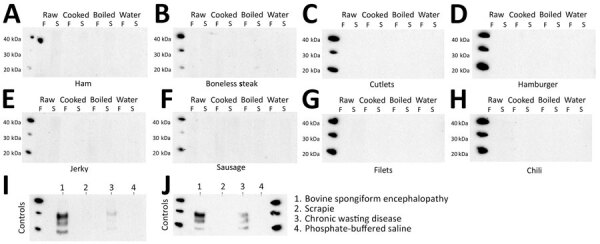
Western blot of PMCA to evaluate zoonotic potential of CWD-containing meats in a study of chronic wasting disease prions in raw, processed, and cooked elk meat. A) Ham; B) boneless steak; C) cutlets; D) hamburger; E) jerky; F) sausage; G) filets; H) chili meat; I) positive control; J) negative control. We evaluated raw, processed, and cooked meat from a CWD-positive elk for the ability to misfold the human prion protein in a single PMCA round. All samples were treated with proteinase K. The analysis depicts samples frozen before PMCA, or sonicated and submitted to PMCA. PBS and scrapie prions were used as negative controls for human PMCA reactions, and BSE and an elk CWD isolates were used as positive controls. Numbers at the left depict molecular weight markers. BSE, bovine spongiform encephalopathy; CWD, chronic wasting disease; F, frozen; PBS, phosphate-buffered saline; PMCA, protein misfolding cyclic amplification; S, sonicated.

Considering the presence of CWD prions in the previously tested edible products, and their persistence after processing and cooking, we evaluated the zoonotic properties of CWD prions by using the PMCA technique. PMCA previously has been reported to be useful in estimating zoonotic potential for multiple animal (i.e., nonhuman) prion isolates (Appendix). Here, we evaluated raw and cooked meats for their potential to template the misfolding of the human PrP^C^ in a single PMCA round to avoid further adaptation of the agent; hence, the goal of our experiment was to mimic initial interspecies transmission events. We specifically used a PMCA protocol optimized for human PrP encoding methionine at position 129. We used that specific human PrP version as PMCA substrate because of its increased susceptibility for misfolding in the presence of BSE prions (Appendix). The results demonstrated that none of the meat samples tested were able to induce conversion of human PrP^C^ to PrP^Sc^, suggesting a limited zoonotic potential for such edible products (Figure 3). Of note, the results were the same regardless of the cooking status of the meat (Figure 3). 

To validate the PMCA method we used for modeling cross-species prion transmission, we incorporated classic BSE and sheep scrapie samples into the experimental protocol. We found PMCA reactions seeded by BSE prions and a CWD isolate were able to induce the misfolding of human PrP^C^, as previously reported (Appendix), but that sheep scrapie was unable to do so.

## Conclusions

Overall, our study results confirm previous reports describing the presence of CWD prions in elk muscles (*13*). The data also demonstrated CWD prion persistence in food products even after processing through different procedures, including the addition of salts, spices, and other edible elements. Of note, our data show that exposure to high temperatures used to cook the meat increased the availability of prions for in vitro amplification. Considering the potential implications in food safety and public health, we believe that the findings described in this study warrant further research. Our results suggest that although the elk meat used in this study resisted different manipulations involved in subsequent consumption by humans, their zoonotic potential was limited. Nevertheless, even though no cases of CWD transmission to human have been reported, the potential for human infection is still unclear and continued monitoring for zoonotic potential is warranted.

Appendix Additional information on chronic wasting disease prions in raw, processed, and cooked elk meat, Texas, USA.

## References

[1] Mould DL, Smith W. The causal agent of scrapie. II. Extraction of the agent from infected goat tissue. J Comp Pathol. 1962;72:106–12. 10.1016/S0368-1742(62)80012-5 14476627

[2] Gambetti P, Kong Q, Zou W, Parchi P, Chen SG. Sporadic and familial CJD: classification and characterisation. Br Med Bull. 2003;66:213–39. 10.1093/bmb/66.1.213 14522861

[3] Bruce ME, Will RG, Ironside JW, McConnell I, Drummond D, Suttie A, et al. Transmissions to mice indicate that ‘new variant’ CJD is caused by the BSE agent. Nature. 1997;389:498–501. 10.1038/39057 9333239

[4] Holt TA, Phillips J. Bovine spongiform encephalopathy. Br Med J (Clin Res Ed). 1988;296:1581–2. 10.1136/bmj.296.6636.1581 3135018 PMC2545961

[5] Miller MW, Williams ES. Chronic wasting disease of cervids. In: Compens RW, Cooper MD, Honjo T, Melchers F, Olsnes S, Vogt PK, editors. Current topics in microbiology and immunology, volume 284. Berlin: Springer-Verlag; 2004. p. 193–214. 10.1007/978-3-662-08441-0_8 15148993

[6] Will RG, Ironside JW, Zeidler M, Cousens SN, Estibeiro K, Alperovitch A, et al. A new variant of Creutzfeldt-Jakob disease in the UK. Lancet. 1996;347:921–5. 10.1016/S0140-6736(96)91412-9 8598754

[7] Cassard H, Torres J-M, Lacroux C, Douet J-Y, Benestad SL, Lantier F, et al. Evidence for zoonotic potential of ovine scrapie prions. Nat Commun. 2014;5:5821. 10.1038/ncomms6821 25510416

[8] Hannaoui S, Zemlyankina I, Chang SC, Arifin MI, Béringue V, McKenzie D, et al. Transmission of cervid prions to humanized mice demonstrates the zoonotic potential of CWD. Acta Neuropathol. 2022;144:767–84. 10.1007/s00401-022-02482-9 35996016 PMC9468132

[9] Race B, Williams K, Chesebro B. Transmission studies of chronic wasting disease to transgenic mice overexpressing human prion protein using the RT-QuIC assay. Vet Res. 2019;50:6. 10.1186/s13567-019-0626-2 30670087 PMC6341683

[10] Li M, Schwabenlander MD, Rowden GR, Schefers JM, Jennelle CS, Carstensen M, et al. RT-QuIC detection of CWD prion seeding activity in white-tailed deer muscle tissues. Sci Rep. 2021;11:16759. 10.1038/s41598-021-96127-8 34408204 PMC8373970

[11] Angers RC, Browning SR, Seward TS, Sigurdson CJ, Miller MW, Hoover EA, et al. Prions in skeletal muscles of deer with chronic wasting disease. Science. 2006;311:1117. 10.1126/science.1122864 16439622

[12] Bravo-Risi F, Soto P, Eckland T, Dittmar R, Ramírez S, Catumbela CSG, et al. Detection of CWD prions in naturally infected white-tailed deer fetuses and gestational tissues by PMCA. Sci Rep. 2021;11:18385. 10.1038/s41598-021-97737-y 34526562 PMC8443553

[13] Spraker TR, Gidlewski T, Powers JG, Nichols TA, Wild MA. Distribution of the misfolded isoform of the prion protein in peripheral tissues and spinal cord of Rocky Mountain elk (*Cervus elaphus nelsoni*) with naturally occurring chronic wasting disease. Vet Pathol. 2023;60:420–33. 10.1177/03009858231173467 37199487

[14] Escobar LE, Pritzkow S, Winter SN, Grear DA, Kirchgessner MS, Dominguez-Villegas E, et al. The ecology of chronic wasting disease in wildlife. Biol Rev Camb Philos Soc. 2020;95:393–408. 10.1111/brv.12568 31750623 PMC7085120

[15] Lonergan SM, Topel DG, Marple DN. Sausage processing and production. In: Lonergan SM, Topel DG, Marple DN, editors. The science of animal growth and meat technology. Cambridge (MA): Elsevier; 2019. p. 229–53.

