# Supplementary material for: Detection of Chronic Wasting Disease Prions in Raw, Processed, and Cooked Elk Meat, Texas, USA
Source: Emerg Infect Dis. 2025 Feb;31(2):363–7. doi: 10.3201/eid3102.240906 (PMC11845151; doi:10.3201/eid3102.240906)
Supplement: Appendix — Additional information on chronic wasting disease prions in raw, processed, and cooked elk meat, Texas, USA. [file 24-0906-Techapp-s1.pdf]

Article DOI: https://doi.org/10.3201/eid3102.240906

EID cannot ensure accessibility for supplementary materials supplied by authors. Readers who have difficulty accessing supplementary content should contact the authors for assistance.

Detection of Chronic Wasting Disease Prions in Raw, Processed, and Cooked Elk Meat, Texas, USA

Appendix

Introduction

Transmissible spongiform encephalopathies (TSEs), also known as prion diseases, are a rare group of fatal neurodegenerative disorders caused by the misfolding of a membrane protein referred to as the cellular prion protein (PrP^C) (*1*). The misfolded, disease-associated prion protein (termed PrP^{Sc}) templates conformational changes on PrP^C that later deposit mostly in the brain and lymphoid tissues of the infected host (*2*). TSEs can affect various animal species, including sheep and goats (*3*), humans (*4*,*5*), cattle (*6*), and cervids (*7*), resulting in diseases known as scrapie, Creutzfeldt-Jakob disease (CJD), bovine spongiform encephalopathy (BSE), and chronic wasting disease (CWD), respectively. TSEs can arise by either sporadic, genetic or infectious mechanisms (*1*).

In the 1990s, several atypical cases of CJD occurred as a result of the ingestion of cattle-derived products infected with BSE. This was later attributed to the emergence of a new human prion strain templated by BSE prions (*8*). Due to this, stringent measures were and are still enacted to prevent the transmission of certain animal TSEs, including BSE and scrapie. These include the removal of infected herds, and the elimination of high-risk tissues (e.g., brain, tonsils, bone marrow) for consumption (*9*). These animal-to-human prion transmission events uncovered the significant threat that BSE pose to public health. Subsequent studies have been conducted to investigate the zoonotic potential of other TSEs, including scrapie, which is recognized to display minimal to no zoonotic potential (*10*), and CWD (*11–15*). CWD affects a diverse range of cervids (*16–21*). The genetic differences and polymorphisms present in CWD-susceptible

animals is thought to contribute to the diversity of prion isolates, also referred to as prion strains (*22*). The latter is relevant, as each prion strain has different potentials to infect other species (*12*). At present, at least 10 different CWD strains have been identified (*22*,*23*), although additional and still unidentified CWD prion strains are expected to exist in both captive and free-ranging cervid populations (*24*). Even when no cases of CWD transmission to human have been reported, the potential for human infection is still unclear, as studies in in vitro systems, animal models, and non-human primates have reported contradictory results (*14*,*15*,*25*–*32*).

Recent studies have raised concerns about the potential for TSEs to be transmitted through other tissues, including those used in the production of processed meats. For instance, experimental exposure of monkeys to infectious prions has shown that these misfolded proteins can be detected in the preclinical stage in tissues such as the tongue, muscle, and heart (*33*). Additionally, CWD prions have been detected in the muscle of both farmed and wild deer (*34*) at concentrations relevant to sustain disease transmission (*35*). Importantly, CWD prions have also been identified in multiple other tissues, such as lymph nodes, spleen, tongue, intestines, adrenal gland, eyes, reproductive tissues, ears, lungs, liver, and more across multiple cervid species (*36*–*38*). This raises concerns about the safety of ingesting processed meats that contain tissues other than skeletal muscle (*39*,*40*) and highlights the need for continued vigilance and research on the transmission risks of TSEs and the development of new preventative and detection measures to ensure the safety of the human food supply. This is relevant considering that products from 7,000 to 15,000 CWD-infected cervids are estimated to be consumed by people per year (*41*).

The first objective of this study was to detect CWD prions in raw and cooked meats, seasoned and unseasoned, prepared from a hunter-harvested elk. The second objective was to assess, by means of a prion amplification method, the potential of the CWD prions contained in these meats to template the misfolding of human PrP^C. In summary, this study provides valuable information regarding the prevalence of CWD prions in cervid-derived human food sources, as well as describing potential methods focused on food safety and estimation of zoonotic risks.

Materials And Methods

Samples

Filets, cutlets, regular spiced meat (jerky), hamburger meat, chili ground meat, ham, sausages, and boneless steak (Appendix Figure 1) were donated by a hunter to Texas Parks and Wildlife Department (TPWD). The meats were prepared from a 5 years old bull that was positive for PrP^{Sc} detection in obex (lymph node was not tested). The date of harvest was 12/10/2020, and the sample was confirmed as CWD positive on 1/8/2021 by immunohistochemistry (IHC). This animal was collected in Medina county, Texas, in a private high fenced hunting ranch. Genotyping identified this animal as an ML heterozygous for position 132 of the prion protein. The meats were made using normal processing procedures by a commercial meat processor. Ground and spiced/smoked meats such as jerky, sausage, and chili, may have included tissues from other animals. Same was expected for ground meats such as hamburgers. However, unground meats such as ham, fillet and boneless steak were obtained purely from the CWD-positive bull. "Ham," or round steak, was prepared from a mix of hindlimb muscles. "Filet" was prepared from backstrap/tenderloin (muscles supporting the spine). The "boneless steak" sample, or sirloin/rump, was obtained from another mix of hindlimb muscles. These samples of processed venison were stored at −20°C until transferred to UTHealth-Houston for analyses. At UTHealth-Houston, samples were also stored at −20°C until used.

Preparation of Samples

Samples of each venison product described above were cut into pieces ≈3 × 2 cm and either processed raw or subjected to one of two different methods of cooking (Figure 1). Specifically, "boiled" meat samples were processed by placing them in a 50 mL falcon tube, covering with distilled water, and microwaving for 1 minute at maximum power. "Grilled" meat samples were processed by wrapping each sample in aluminum foil and placing them on a hot plate at 100°C for 7 minutes. The external and internal appearance of "grilled" meat samples gave the appearance of a cooking point of medium-well. The internal temperatures reached by three different meat types (hamburger, sausage, and boneless steak) are described in Appendix Table. Cooked and raw meats were homogenized at 20% weight/volume (w/v) in PBS containing a cocktail of protease inhibitors without EDTA (Roche, Basel, Switzerland). The

homogenization was conducted in a Precellys 24 dual homogenizer (Bertin instruments, MP biomedicals, Irvine, CA, USA).

Protein Misfolding Cyclic Amplification (PMCA) using Elk Substrate

The PMCA procedure was conducted following the detailed protocol described in our previously published reports (*42*), with specific adjustments made for CWD (*43*). Briefly, brains from Tg5037 (*18*) mice (expressing the elk prion protein sequence) were homogenized at a concentration of 10% w/v in PMCA conversion buffer, containing PBS, 150 mM NaCl, 1% Triton X-100, and a protease inhibitors cocktail (Roche, Basel, Switzerland). This homogenate was then centrifuged at 805 x *g* for 1 min at 4°C and the supernatant was collected to generate the PMCA substrate. These homogenates were supplemented with digitonin and EDTA at final concentrations of 0.025 % and 6 mM, respectively. Aliquots of 90 µL of this PMCA substrate were mixed with either 10 µL of raw, boiled, or grilled meat homogenates, or water from the boiling procedure, and subjected to 144 cycles of incubation and sonication, with each PMCA cycle consisting of 29 minutes and 40 seconds of incubation, and 20 seconds of sonication. The resulting samples were then subjected to two additional rounds of PMCA, with 96 cycles in each round, by mixing 10 µL of the PMCA products from each round with fresh PMCA substrate (90 µL). The purpose of these additional rounds was to increase the detection of CWD prions in the tested samples (Appendix Figure 2). Each PMCA set, including 36 reactions, was controlled by adding tubes containing serial dilutions of a CWD brain of known PMCA activity (Appendix Figure 2), as well four unseeded reactions. Each specimen was tested in duplicate by two different investigators that were blinded to the status of the samples. The CWD statuses of the samples were evaluated at all the PMCA rounds. A sample was considered as CWD positive if at least one of the two replicates provided positive results.

Evaluation of Zoonotic Potential of CWD-Positive Meat using PMCA

It is important to note that modifications in the PMCA protocol are usually made to optimize the misfolding of prion proteins from different animal species. For that reason, a different PMCA protocol was used to assess the zoonotic potential of the meats tested in this study. Specifically, transgenic mouse brains expressing the human version of the prion protein (129MM (*44*)) were homogenized in conversion buffer by using a glass-on-glass manual grinder and a conversion buffer made of PBS, 150 mM NaCl, 1% Triton X-100, and a complete protease inhibitor cocktail (cOmplete; Roche, Mannheim, Germany) to obtain a final 10% w/v solution.

The homogenized tissue was cleared by using centrifugation (40 seconds at 805 x *g*), the supernatant (*i.e.*, human PMCA substrate) was aliquoted in 1.5 mL tubes, and stored at −80°C until used. The in vitro prion amplification was conducted in a programmable Q-700 sonicator attached to a microplate titanium horn. Low molecular weight heparin at 100 µg/mL and EDTA was included in all the 1.5 mL tubes to a final concentration of 6 mM each. We mixed elk meat homogenates with aliquots of human PMCA substrate in a final volume of 120 µL in PCR tubes at a 1/10 dilution. BSE, elk CWD (homozygous for methionine at position 132 of the prion protein (*45*)) and scrapie of (ARQ/ARQ genotype) were all included in the reaction at a dilution 1/10, except for BSE which was used at a dilution 1/5. To perform a comparison between samples before and after the amplification procedure, we took 19 µL of each reaction mixture before the serial cycles of sonication and incubation. Each cycle consisted of 20 s sonication (at an amplitude of 33, wattage range 280–300) followed by 29 min and 40 s incubation; we repeated this procedure 96 times (48 h). These experiments were performed at least twice, yielding consistent results.

Proteinase K (PK) Digestion and Western Blotting for Elk PMCA Products

To visualize the presence of PrP^{Sc} in elk PMCA products, samples were treated with PK (Sigma-Aldrich, Saint Louis, MO, USA) at a final concentration of 100 µg/mL. PK digestion was carried out at 37°C with shaking for 80 minutes. PK reactions were stopped by adding LDS sample buffer and heating at 95°C for 10 minutes. After digestion, 20 µL of sample were fractionated on NuPAGE 12% Bis-Tris gels (Invitrogen, Carlsbad, CA, USA) and transferred to nitrocellulose membranes (GE Healthcare Amersham, Chicago, IL, USA). The membranes were then blocked using a 5% w/v non-fat milk solution and probed with a monoclonal Bar224 antibody (Bertin Corp, Rockville, MD, USA) at a dilution of 1:10,000. After washing, the membranes were incubated with a secondary antibody, polyclonal anti-mouse IgG (whole molecule) - peroxidase antibody produced in sheep (Sigma-Aldrich, Saint Louis, MO, USA), at a dilution of 1:3,000. The membranes were washed again and developed using an ECL kit (GE Healthcare Amersham, Chicago, IL, USA) following the manufacturer's recommendations. As mentioned, the presence of CWD prions in a given sample was assumed if at least one of the replicates resulted in positive detection, considering that all unseeded PMCA reactions in our study yielded negative PrP^{Sc} signals.

PK Digestion and Western Blotting for Human PMCA Products

The PMCA products were evaluated by western blot after PK treatment. Nineteen μL of each sample were incubated with PK at a final concentration of 50 μg/mL for 1 h at 37°C in a standard thermoblock. Before electrophoretic fractionation, the samples were mixed with 10 μL 4x NuPAGE buffer (Invitrogen, Carlsbad, CA, USA) and boiled at 100°C for 10 min. The samples were then loaded on NuPAGE Novex (Fisher Scientific; Loughborough, UK) 10% Bis-Tris gels (1.0 mm, 10 wells) and subjected to electrophoresis at 200 V for 55 min. The proteins were transferred to a polyvineladine difluoride membrane by using by using 800 mA for 60 min. Membranes were blocked with 2% milk for 1 h. Human prion protein conversion was determined on the basis of the specific immune-reactivity of the 3F4 monoclonal antibody diluted 1:10,000 (Millipore; Watford, UK) (*46*). To detect the original PrP^{Sc} input, the same membranes were stripped (Thermo-Fisher, Bleiswijk, Netherlands) and reprobed with the 6H4 antibody diluted 1:40,000 (Prionics, Schlieren, Switzerland). Membranes were then incubated with ECL anti-mouse IgG, peroxide-linked-species-specific F(ab')2 fragment from sheep (GE Lifecare Health Sciences; Little Chalfont, UK) diluted 1/25,000. The membranes were developed by chemiluminescent detection using ECL Prime (GE Healthcare Life Sciences) and acquired digital images by using an XRS Bio-Rad system (Bio-Rad Laboratories, Hercules, CA, USA) with a CCD camera.

Results

Detection of CWD Prions in Raw and Cooked Unprocessed and Processed Meat

Different unprocessed and processed meats were obtained from a hunter-harvested, CWD-infected elk. The meats and meat-derived products used in this study included filets, jerky steak cuts, hamburger meat, chili meat, sausage, ham, cutlets and boneless steaks (Appendix Figure 1). These raw meat samples were tested for the presence of CWD prions using the PMCA technique in elk substrate as described in Figure 1. The elk PMCA substrate was selected (*18*) to maintain the prion protein sequence homology between PrP^{C} and the suspected PrP^{Sc} present in meats. In a first PMCA round, prion detection was negative for most of the raw meat samples, with the exception of the boneless steak that provided a positive PMCA signal in one of the replicates (Figure 2). To increase the sensitivity of prion detection, two additional PMCA rounds were performed (Appendix Figure 2). In a second PMCA round, positive signals were observed

for additional specimens, including sausages and cutlets (Figure 2). Additionally, the jerky sample provided CWD-prion signals in a third PMCA round (Figure 2). No other samples were tested as PMCA positive in this analysis.

To test the persistence of CWD prions in these meat products, different pieces of the meat types mentioned above were grilled, mimicking a “medium-well” cooking status considering their external and internal appearance, in addition to their internal temperatures (Appendix Table 1). Surprisingly, grilling substantially increased the detection of prions by PMCA considering that five sample types (hamburger, chili, ham, cutlets and boneless steak meats) were PMCA-positive in a first round (Figure 2). A positive PMCA signal was observed for jerky meats in a second PMCA round, as well as an increase in the number of positive replicates for the samples detected in the first round. At the third PMCA round, all grilled and boiled meats were positive for CWD prion detection in at least one replicate, strongly suggesting that grilling increased the availability of CWD prions for in vitro prion amplification (Figure 2).

Similar results as those noted for the grilled meats were observed when different cuts of the same specimens were boiled (Figure 2). Specifically, CWD prions were detected in chili, ham and boneless steak samples in a first PMCA round, with the latter specimen providing positive results in both replicates. Fillets, and cutlets were also identified as CWD positive in a second PMCA round. By the third PMCA round, all specimens provided positive PMCA outcomes in at least one of the replicates. Interestingly, CWD prions were also detected in the water used to boil the chili meat samples (first PMCA round) and fillets (third PMCA round) (Figure 2 and Appendix Figure 3). The purpose of performing these serial PMCA rounds was to obtain a semiquantitative estimation of the prion content in each specimen and procedure. This is based in the well described fact that prion detection increases by performing serial PMCA rounds (Appendix Figure 2).

Overall, these results further confirm previous studies demonstrating the presence of CWD prions in elk muscles (*37*). These data also demonstrate the CWD-prion persistence in these food products even after processed through different procedures including the addition of salts, spices and other edible elements. Importantly, our data show that exposure to high temperatures used to cook these meats increase the availability of prions for in vitro prion replication.

Exploring the Zoonotic Potential of CWD-Contaminated Elk Meat and Meat-Derived Products using PMCA

Considering the presence of CWD prions in the previously tested edible products, and their persistence after processing and cooking, we evaluated their zoonotic properties using the PMCA technique. PMCA has been previously reported to be useful in estimating zoonotic potentials for multiple animal (non-human) prion isolates (*44*,*46*). Here, raw and cooked meats were evaluated for their potential to template the misfolding of the human PrP^C in a single PMCA round to avoid further adaptation of the agent (*ergo*, our experiment has the goal to mimic initial inter-species transmission events). We specifically used a PMCA protocol optimized for human PrP encoding methionine at position 129. This specific human PrP version was used as PMCA substrate considering its increased susceptibility for misfolding in the presence of BSE prions (*47–49*).

The results demonstrate that none of the meat samples tested in these studies were able to induce the conversion of human PrP^C to PrP^{Sc}, suggesting a limited zoonotic potential for these edible products (Figure 3). Importantly, the results were the same regardless of the cooking status of the meats (Figure 3). To validate the PMCA method employed in this study for modeling cross-species prion transmission, classical BSE and sheep scrapie samples were incorporated into the experimental protocol. These prion strains were used in this system to control this technique for the evaluation of zoonotic risks (*45*). PMCA reactions seeded by BSE prions and a CWD isolate were able to induce the misfolding of human PrP^C, as previously reported (*45*). The data presented in Appendix Figure 4 further demonstrate that the signals induced by BSE and CWD prions are due to in vitro amplification of protein misfolding and not to the original inocula. Unfortunately, the brain from the elk from which meats were collected was not available so it was not possible to include it in these analyses.

In summary, the data presented in Figure 3 suggest that although the elk meats used in this study resist different manipulations involved in their subsequent consumption by humans, their zoonotic potential is limited.

Discussion

One of the primary concerns surrounding CWD lies in its unknow zoonotic potential (*12*,*13*,*50*,*51*). This concern is valid, given the historical context of the BSE epidemic (*52*). Despite the extensive prevalence of infected cattle in United Kingdom, the clinical evidence of BSE transmission to humans (known as vCJD) was lower than anticipated (*53*). Nevertheless, more recent data suggest that the number of asymptomatic individuals was considerable higher (*54*). Although no cases of CWD transmission to humans have been reported at present, epidemiologic studies have been focused only on clinical cases and not subclinical infections. Regardless, experimental data using animal models (*14*,*29*,*31*,*55*,*56*) and in vitro systems (*15*,*46*,*57*) suggest that although CWD can template the misfolding of human PrP^C, there is a considerable barrier for these events to occur. Nevertheless, the odds for zoonotic transmissions are expected to increase if CWD-infected materials enter the human food supply. Currently, testing meat products is not mandatory and the approximated quantities of CWD contaminated materials consumed by people is estimated to be considerable.

In this study, we describe the presence of CWD prions in a variety of unprocessed and processed meats derived from a preclinical (not displaying clinical signs), CWD-infected elk. Regardless the use of high concentrations of salt, spices, and other food ingredients, prions were detected by PMCA in products such as sausages, jerky and chili meats. To further explore the availability of prions in these meats, samples were cooked by either grilling or boiling. Surprisingly, we found that cooking increased the availability of prions for in vitro amplification in all sample types. This unexpected result could be explained by the fact that heat helps in disrupting tissue structures trapping prions even after homogenization. Unfortunately, our in vitro analysis does not allow us to estimate whether this increase in PMCA detection after cooking has any meaningful effect in inter-individual transmissions. This is relevant considering a previous report describing that hot dogs contaminated with 263K prions (a laboratory generated prion strain adapted in Syrian hamsters) showed substantial reduction of infectivity after being subjected to ultra-high pressure (*58*). Future experiments in animal models may be able to provide additional information regarding this relevant question.

We also found that the CWD-prions present in these meats were unable to template the misfolding of human PrP^C to PrP^{Sc}, suggesting a limited zoonotic potential for these edible

products. Nevertheless, these results need to be treated with caution considering multiple factors. First, it is unknown whether higher quantities of the CWD prions contained in other meat samples will be able to induce the misfolding of the human prion protein. Although this is a possibility, the PMCA technique is not designed to evaluate this scenario and this paradigm must be explored using animal models. Second, the extent of prion infection in the original elk was unknown. This is an important consideration as the levels of prion accumulation, especially in peripheral tissues, depends (among other factors) on the prion incubation periods. In that sense, it is uncertain whether clinical animals, or subjects at later stages of the preclinical stages of the disease, will contain higher quantities of CWD prions in muscles or other tissues relevant for human consumption. Third, a single CWD prion infected animal was evaluated in this study. This animal likely contained specific prion conformers (strains) considering its polymorphic variation at position 132 of the prion protein. It is well documented that polymorphic variation favors the appearance of different prion strains. In turn, these may manifest with diverse distribution across different tissues (*59*,*60*) and host ranges (*60–62*). Considering this, future studies analyzing additional contaminated meats prepared from animals carrying different PrP polymorphisms or prion strains are warranted considering both in vitro screening practices and in vivo evaluations of disease transmission.

Conclusions and Future Directions

Although the results provided here are of relevance for animal production and public health, this must be considered as an initial study involving food safety and CWD. As mentioned above, this study raised multiple questions. Among them, what is the relationship between the biodistribution of prions in cervid-edible parts at different stages of the disease? How variable is the prion distribution in diverse muscles across animal species infected by different prion strains? Are hunters or scavenger species (i.e., cougars, wild pigs) susceptible to prions present in muscles? Are the prion quantities present in muscles at quantities capable of sustaining inter-species prion transmission? All these questions are matters of future research that are warranted considering the results presented in this report.

References

1. Liemann S, Glockshuber R. Transmissible spongiform encephalopathies. Biochem Biophys Res Commun. 1998;250:187–93. PubMed https://doi.org/10.1006/bbrc.1998.9169

2. Sejvar JJ, Schonberger LB, Belay ED. Transmissible spongiform encephalopathies. J Am Vet Med Assoc. 2008;233:1705–12. PubMed https://doi.org/10.2460/javma.233.11.1705

3. Mould DL, Smith W. The causal agent of scrapie. II. Extraction of the agent from infected goat tissue. J Comp Pathol. 1962;72:106–12. PubMed https://doi.org/10.1016/S0368-1742(62)80012-5

4. Gambetti P, Kong Q, Zou W, Parchi P, Chen SG. Sporadic and familial CJD: classification and characterisation. Br Med Bull. 2003;66:213–39. PubMed https://doi.org/10.1093/bmb/66.1.213

5. Bruce ME, Will RG, Ironside JW, McConnell I, Drummond D, Suttie A, et al. Transmissions to mice indicate that 'new variant' CJD is caused by the BSE agent. Nature. 1997;389:498–501. PubMed https://doi.org/10.1038/39057

6. Holt TA, Phillips J. Bovine spongiform encephalopathy. Br Med J (Clin Res Ed). 1988;296:1581–2. PubMed https://doi.org/10.1136/bmj.296.6636.1581

7. Miller MW, Williams ES. Chronic wasting disease of cervids. In: Compens RW, Cooper MD, Honjo T, Melchers F, Olsnes S, Vogt PK, editors. Current topics in microbiology and immunology, vol 284. Berlin: Springer-Verlag; 2004. p. 193–214.

8. Will RG, Ironside JW, Zeidler M, Cousens SN, Estibeiro K, Alperovitch A, et al. A new variant of Creutzfeldt-Jakob disease in the UK. Lancet. 1996;347:921–5. PubMed https://doi.org/10.1016/S0140-6736(96)91412-9

9. US Department of Agriculture. Prohibition of the use of specified risk materials for human food and requirements for the disposition of non-ambulatory disabled cattle [2025 Jan 14]. https://www.fsis.usda.gov/policy/federal-register-rulemaking/federal-register-rules/prohibition-use-specified-risk-materials

10. Peden AH, Suleiman S, Barria MA. Understanding intra-species and inter-species rrion conversion and zoonotic potential using protein misfolding cyclic amplification. Front Aging Neurosci. 2021;13:716452. PubMed https://doi.org/10.3389/fnagi.2021.716452

11. Cassard H, Torres JM, Lacroux C, Douet JY, Benestad SL, Lantier F, et al. Evidence for zoonotic potential of ovine scrapie prions. Nat Commun. 2014;5:5821. PubMed https://doi.org/10.1038/ncomms6821

12. Saunders SE, Bartelt-Hunt SL, Bartz JC. Occurrence, transmission, and zoonotic potential of chronic wasting disease. Emerg Infect Dis. 2012;18:369–76. PubMed https://doi.org/10.3201/eid1803.110685

13. Pritzkow S. Transmission, strain diversity, and zoonotic potential of chronic wasting disease. Viruses. 2022;14:1390. PubMed https://doi.org/10.3390/v14071390

14. Hannaoui S, Zemlyankina I, Chang SC, Arifin MI, Béringue V, McKenzie D, et al. Transmission of cervid prions to humanized mice demonstrates the zoonotic potential of CWD. Acta Neuropathol. 2022;144:767–84. PubMed https://doi.org/10.1007/s00401-022-02482-9

15. Pritzkow S, Gorski D, Ramirez F, Telling GC, Benestad SL, Soto C. North American and Norwegian chronic wasting disease prions exhibit different potential for interspecies transmission and zoonotic risk. J Infect Dis. 2022;225:542–51. PubMed https://doi.org/10.1093/infdis/jiab385

16. Moore SJ, Kunkle R, Greenlee MH, Nicholson E, Richt J, Hamir A, et al. Horizontal transmission of chronic wasting disease in reindeer. Emerg Infect Dis. 2016;22:2142–5. PubMed https://doi.org/10.3201/eid2212.160635

17. Pirisinu L, Tran L, Chiappini B, Vanni I, Di Bari MA, Vaccari G, et al. Novel type of chronic wasting disease detected in moose (*Alces alces*), Norway. Emerg Infect Dis. 2018;24:2210–8. PubMed https://doi.org/10.3201/eid2412.180702

18. Angers RC, Seward TS, Napier D, Green M, Hoover E, Spraker T, et al. Chronic wasting disease prions in elk antler velvet. Emerg Infect Dis. 2009;15:696–703. PubMed https://doi.org/10.3201/eid1505.081458

19. Gossert AD, Bonjour S, Lysek DA, Fiorito F, Wüthrich K. Prion protein NMR structures of elk and of mouse/elk hybrids. Proc Natl Acad Sci U S A. 2005;102:646–50. PubMed https://doi.org/10.1073/pnas.0409008102

20. Tranulis MA, Gavier-Widén D, Våge J, Nöremark M, Korpenfelt SL, Hautaniemi M, et al. Chronic wasting disease in Europe: new strains on the horizon. Acta Vet Scand. 2021;63:48. PubMed https://doi.org/10.1186/s13028-021-00606-x

21. Duque Velásquez C, Kim C, Herbst A, Daude N, Garza MC, Wille H, et al. Deer prion proteins modulate the emergence and adaptation of chronic wasting disease strains. J Virol. 2015;89:12362–73. PubMed https://doi.org/10.1128/JVI.02010-15

22. Otero A, Duque Velasquez C, McKenzie D, Aiken J. Emergence of CWD strains. Cell Tissue Res. 2023;392:135–48. PubMed https://doi.org/10.1007/s00441-022-03688-9

23. Sun JL, Kim S, Crowell J, Webster BK, Raisley EK, Lowe DC, et al. Novel prion strain as cause of chronic wasting disease in a moose, Finland. Emerg Infect Dis. 2023;29:323–32. PubMed https://doi.org/10.3201/eid2902.220882

24. Benavente R, Reed JH, Lockwood M, Morales R. PMCA screening of retropharyngeal lymph nodes in white-tailed deer and comparisons with ELISA and IHC. Sci Rep. 2023;13:20171. PubMed https://doi.org/10.1038/s41598-023-47105-9

25. Belay ED, Maddox RA, Williams ES, Miller MW, Gambetti P, Schonberger LB. Chronic wasting disease and potential transmission to humans. Emerg Infect Dis. 2004;10:977–84. PubMed https://doi.org/10.3201/eid1006.031082

26. Nemani SK, Myskiw JL, Lamoureux L, Booth SA, Sim VL. Exposure risk of chronic wasting disease in humans. Viruses. 2020;12:1454. PubMed https://doi.org/10.3390/v12121454

27. Wadsworth JDF, Joiner S, Linehan JM, Jack K, Al-Doujaily H, Costa H, et al. Humanized transgenic mice are resistant to chronic wasting disease prions from Norwegian reindeer and moose. J Infect Dis. 2022;226:933–7. PubMed https://doi.org/10.1093/infdis/jiab033

28. Kong Q, Huang S, Zou W, Vanegas D, Wang M, Wu D, et al. Chronic wasting disease of elk: transmissibility to humans examined by transgenic mouse models. J Neurosci. 2005;25:7944–9. PubMed https://doi.org/10.1523/JNEUROSCI.2467-05.2005

29. Wang Z, Qin K, Camacho MV, Cali I, Yuan J, Shen P, et al. Generation of human chronic wasting disease in transgenic mice. Acta Neuropathol Commun. 2021;9:158. PubMed https://doi.org/10.1186/s40478-021-01262-y

30. Sandberg MK, Al-Doujaily H, Sigurdson CJ, Glatzel M, O'Malley C, Powell C, et al. Chronic wasting disease prions are not transmissible to transgenic mice overexpressing human prion protein. J Gen Virol. 2010;91:2651–7. PubMed https://doi.org/10.1099/vir.0.024380-0

31. Race B, Williams K, Chesebro B. Transmission studies of chronic wasting disease to transgenic mice overexpressing human prion protein using the RT-QuIC assay. Vet Res. 2019;50:6. PubMed https://doi.org/10.1186/s13567-019-0626-2

32. Wilson R, Plinston C, Hunter N, Casalone C, Corona C, Tagliavini F, et al. Chronic wasting disease and atypical forms of bovine spongiform encephalopathy and scrapie are not transmissible to mice expressing wild-type levels of human prion protein. J Gen Virol. 2012;93:1624–9. PubMed https://doi.org/10.1099/vir.0.042507-0

33. Krasemann S, Neumann M, Geissen M, Bodemer W, Kaup FJ, Schulz-Schaeffer W, et al. Preclinical deposition of pathological prion protein in muscle of experimentally infected primates. PLoS One. 2010;5:e13906. PubMed https://doi.org/10.1371/journal.pone.0013906

34. Li M, Schwabenlander MD, Rowden GR, Schefers JM, Jennelle CS, Carstensen M, et al. RT-QuIC detection of CWD prion seeding activity in white-tailed deer muscle tissues. Sci Rep. 2021;11:16759. PubMed https://doi.org/10.1038/s41598-021-96127-8

35. Angers RC, Browning SR, Seward TS, Sigurdson CJ, Miller MW, Hoover EA, et al. Prions in skeletal muscles of deer with chronic wasting disease. Science. 2006;311:1117. PubMed https://doi.org/10.1126/science.1122864

36. Bravo-Risi F, Soto P, Eckland T, Dittmar R, Ramírez S, Catumbela CSG, et al. Detection of CWD prions in naturally infected white-tailed deer fetuses and gestational tissues by PMCA. Sci Rep. 2021;11:18385. PubMed https://doi.org/10.1038/s41598-021-97737-y

37. Spraker TR, Gidlewski T, Powers JG, Nichols TA, Wild MA. Distribution of the misfolded isoform of the prion protein in peripheral tissues and spinal cord of Rocky Mountain elk (*Cervus elaphus nelsoni*) with naturally occurring chronic wasting disease. Vet Pathol. 2023;60:420–33. PubMed https://doi.org/10.1177/03009858231173467

38. Escobar LE, Pritzkow S, Winter SN, Grear DA, Kirchgessner MS, Dominguez-Villegas E, et al. The ecology of chronic wasting disease in wildlife. Biol Rev Camb Philos Soc. 2020;95:393–408. PubMed https://doi.org/10.1111/brv.12568

39. Lonergan SM, Topel DG, Marple DN. Sausage processing and production. In: Lonergan SM, Topel DG, Marple DN, editors. The science of animal growth and meat technology. Cambridge (MA): Elsevier; 2019. p. 229–253.

40. Food and Agriculture Organization of the United Nations. Sausage raw materials [cited 2023 May 9]. https://www.fao.org/3/x6556e/X6556E02.htm

41. Geist V, Clausen D, Crichton V, Rowledge D. The challenge of CWD: insidious and dire. Calgary (AB): Alliance for Public Wildlife; 2017.

42. Morales R, Duran-Aniotz C, Diaz-Espinoza R, Camacho MV, Soto C. Protein misfolding cyclic amplification of infectious prions. Nat Protoc. 2012;7:1397–409. PubMed https://doi.org/10.1038/nprot.2012.067

43. Kramm C, Soto P, Nichols TA, Morales R. Chronic wasting disease (CWD) prion detection in blood from pre-symptomatic white-tailed deer harboring *PRNP* polymorphic variants. Sci Rep. 2020;10:19763. PubMed https://doi.org/10.1038/s41598-020-75681-7

44. Bishop MT, Hart P, Aitchison L, Baybutt HN, Plinston C, Thomson V, et al. Predicting susceptibility and incubation time of human-to-human transmission of vCJD. Lancet Neurol. 2006;5:393–8. PubMed https://doi.org/10.1016/S1474-4422(06)70413-6

45. Barria MA, Balachandran A, Morita M, Kitamoto T, Barron R, Manson J, et al. Molecular barriers to zoonotic transmission of prions. Emerg Infect Dis. 2014;20:88–97. PubMed https://doi.org/10.3201/eid2001.130858

46. Barria MA, Libori A, Mitchell G, Head MW. Susceptibility of human prion protein to conversion by chronic wasting disease prions. Emerg Infect Dis. 2018;24:1482–9. PubMed https://doi.org/10.3201/eid2408.161888

47. Asante EA, Linehan JM, Desbruslais M, Joiner S, Gowland I, Wood AL, et al. BSE prions propagate as either variant CJD-like or sporadic CJD-like prion strains in transgenic mice expressing human prion protein. EMBO J. 2002;21:6358–66. PubMed https://doi.org/10.1093/emboj/cdf653

48. Collinge J, Beck J, Campbell T, Estibeiro K, Will RG. Prion protein gene analysis in new variant cases of Creutzfeldt-Jakob disease. Lancet. 1996;348:56. PubMed https://doi.org/10.1016/S0140-6736(05)64378-4

49. Zeidler M, Stewart G, Cousens SN, Estibeiro K, Will RG. Codon 129 genotype and new variant CJD. Lancet. 1997;350:668. PubMed https://doi.org/10.1016/S0140-6736(05)63366-1

50. Otero A, Velásquez CD, Aiken J, McKenzie D. Chronic wasting disease: a cervid prion infection looming to spillover. Vet Res. 2021;52:115. PubMed https://doi.org/10.1186/s13567-021-00986-y

51. Tranulis MA, Tryland M. The zoonotic potential of chronic wasting disease–a review. Foods. 2023;12:824. PubMed https://doi.org/10.3390/foods12040824

52. Collee JG, Bradley R. BSE: a decade on–Part I. Lancet. 1997;349:636–41. PubMed https://doi.org/10.1016/S0140-6736(96)01310-4

53. Jackson GS, Burk-Rafel J, Edgeworth JA, Sicilia A, Abdilahi S, Korteweg J, et al. Population screening for variant Creutzfeldt-Jakob disease using a novel blood test: diagnostic accuracy and feasibility study. JAMA Neurol. 2014;71:421–8. PubMed https://doi.org/10.1001/jamaneurol.2013.6001

54. Gill ON, Spencer Y, Richard-Loendt A, Kelly C, Dabaghian R, Boyes L, et al. Prevalent abnormal prion protein in human appendixes after bovine spongiform encephalopathy epizootic: large scale survey. BMJ. 2013;347(oct15 5):f5675. PubMed https://doi.org/10.1136/bmj.f5675

55. Kong Q, Huang S, Zou W, Vanegas D, Wang M, Wu D, et al. Chronic wasting disease of elk: transmissibility to humans examined by transgenic mouse models. J Neurosci. 2005;25:7944–9. PubMed https://doi.org/10.1523/JNEUROSCI.2467-05.2005

56. Race B, Meade-White KD, Phillips K, Striebel J, Race R, Chesebro B. Chronic wasting disease agents in nonhuman primates. Emerg Infect Dis. 2014;20:833–7. PubMed https://doi.org/10.3201/eid2005.130778

57. Li L, Coulthart MB, Balachandran A, Chakrabartty A, Cashman NR. Species barriers for chronic wasting disease by in vitro conversion of prion protein. Biochem Biophys Res Commun. 2007;364:796–800. PubMed https://doi.org/10.1016/j.bbrc.2007.10.087

58. Brown P, Meyer R, Cardone F, Pocchiari M. Ultra-high-pressure inactivation of prion infectivity in processed meat: a practical method to prevent human infection. Proc Natl Acad Sci U S A. 2003;100:6093–7. PubMed https://doi.org/10.1073/pnas.1031826100

59. Morales R, Abid K, Soto C. The prion strain phenomenon: molecular basis and unprecedented features. Biochim Biophys Acta. 2007;1772:681–91. PubMed https://doi.org/10.1016/j.bbadis.2006.12.006

60. Bartz JC. Prion strain diversity. Cold Spring Harb Perspect Med. 2016;6:a024349. PubMed https://doi.org/10.1101/cshperspect.a024349

61. Herbst A, Velásquez CD, Triscott E, Aiken JM, McKenzie D. Chronic wasting disease prion strain emergence and host range expansion. Emerg Infect Dis. 2017;23:1598–600. PubMed https://doi.org/10.3201/eid2309.161474

62. Manka SW, Wenborn A, Betts J, Joiner S, Saibil HR, Collinge J, et al. A structural basis for prion strain diversity. Nat Chem Biol. 2023;19:607–13. PubMed https://doi.org/10.1038/s41589-022-01229-7

Appendix Table. Internal temperatures of different elk meat products after being used in two cooking procedures

	Internal temperature, °C	
Meat type	Boiled	Grilled
Burger	92.8	71.9
Sausage	98.6	67.0
Boneless steak	75.7	74.7

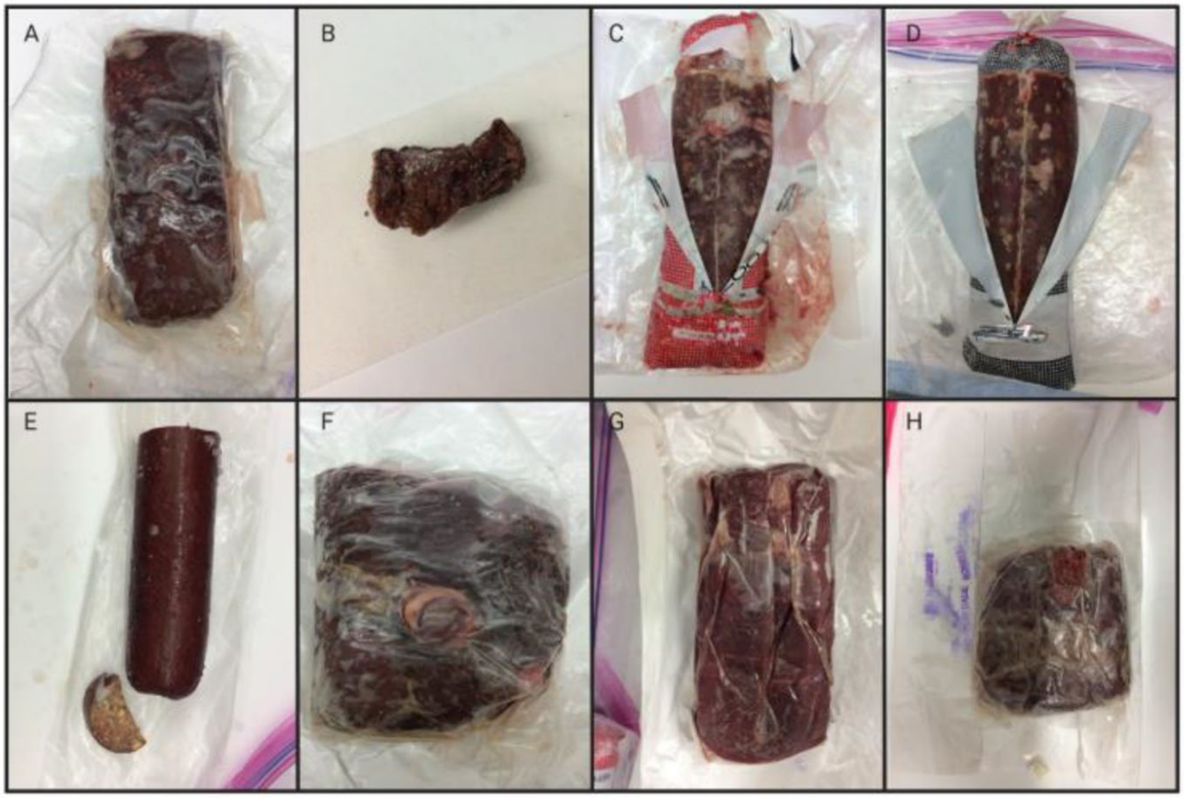

Appendix Figure 1. Photographs of unprocessed and processed elk meat products analyzed in this study. A) Filets; B) jerky elk meat; C) hamburger ground meat; D) chili seasoned meat; E) sausage; F) ham steak; G) cutlets; and H) boneless steak.

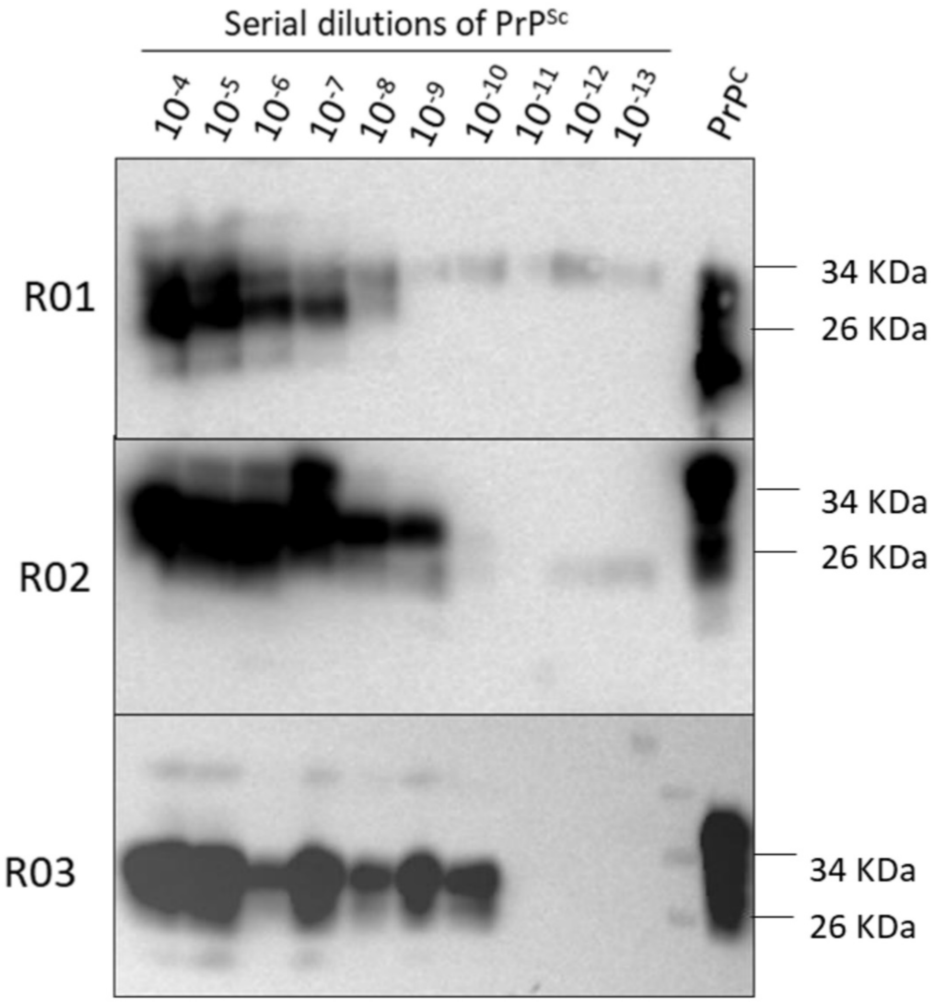

Appendix Figure 2. Serial PMCA of a CWD infected brain of known seeding activity. This panel is representative of the positive control used in all our PMCA reactions. It corresponds to a serially diluted (10^{-4} to 10^{-13}) brain extract from an experimentally CWD infected white-tailed deer that was subjected to 3 PMCA rounds (R01 – R03). All samples were treated with PK, except for PrP^C, which was used as a control of antibody reactivity and molecular weight mobility. Numbers at the right represent molecular weights.

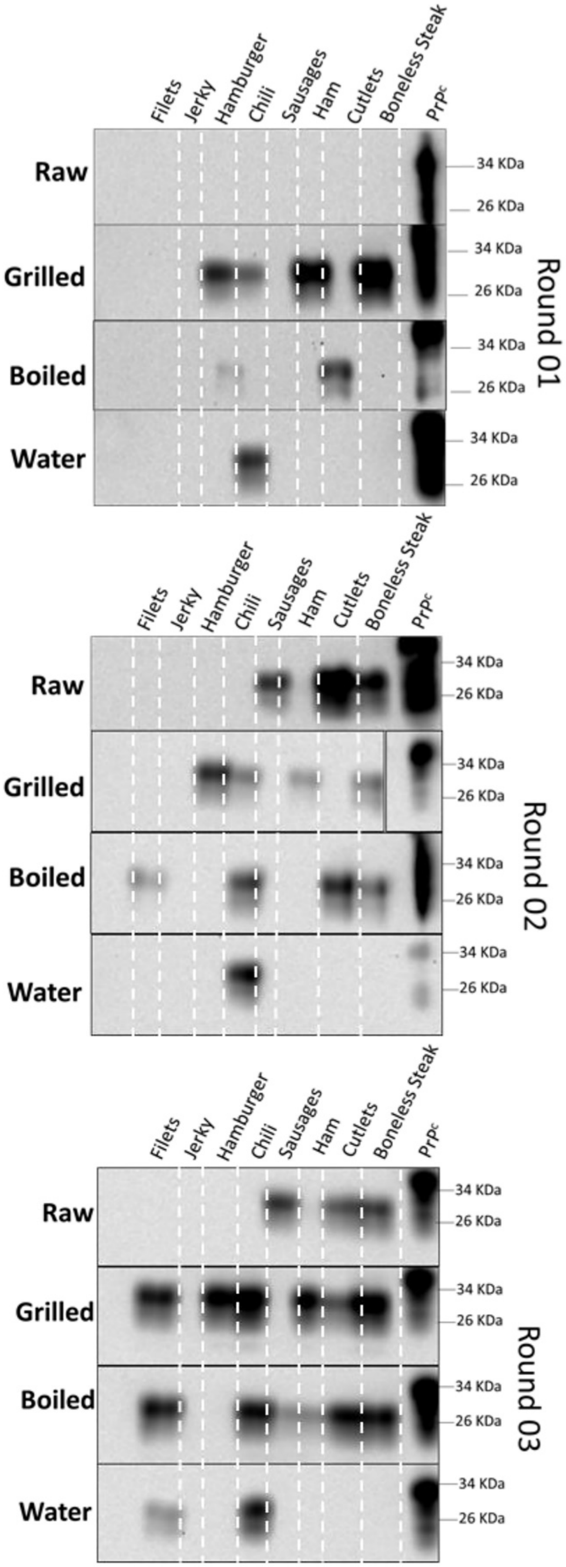

Appendix Figure 3. Marked sample lines for Western blots from Figure 2 in the main text.

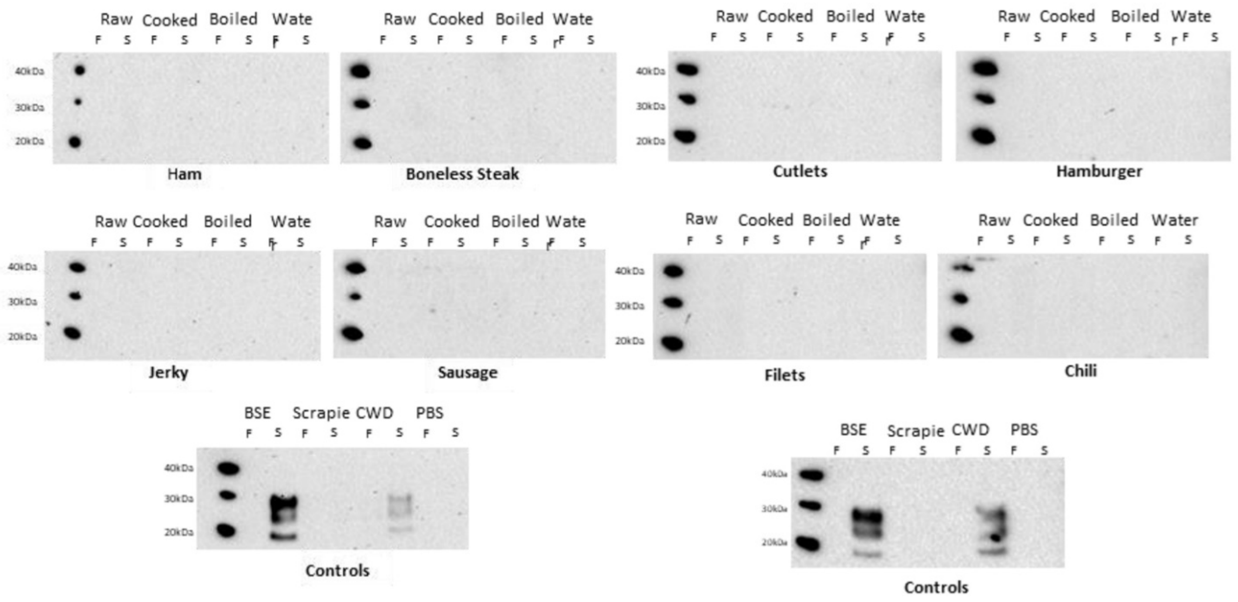

Appendix Figure 4. Western blots to evaluate prion presence in sample-substrate mixtures before PMCA. The same membrane depicted in Figure 3 was probed with the 6H4 antibody recognizing cattle, human, ovine, and cervid PrP sequences. The panels depict the absence of prion detection in all mixture before PMCA (F), demonstrating that PMCA products (S) correspond to in vitro generated prions. Numbers at the right depict molecular weight markers. PMCA, protein misfolding cyclic amplification; PrP, prion protein.
